# Oxytocin as the Neurobiological Basis of Synchronization: A Research Proposal in Psychotherapy Settings

**DOI:** 10.3389/fpsyg.2021.628011

**Published:** 2021-07-09

**Authors:** Arianna Palmieri, Emanuele Pick, Ariella Grossman-Giron, Dana Tzur Bitan

**Affiliations:** ^1^Department of Philosophy, Sociology, Education, and Applied Psychology, University of Padova, Padua, Italy; ^2^Padova Neuroscience Centre, University of Padova, Padua, Italy; ^3^Department of Behavioral Sciences, Ariel University, Ariel, Israel; ^4^Shalvata Mental Health Center, Affiliated With the Sackler School of Medicine, Tel Aviv University, Tel Aviv, Israel

**Keywords:** physiological synchronization, electrodermal activity, oxytocin, social engagement, psychotherapy

## Introduction

Synchronization of physiological signals between individuals seems to originate from intra-uterine life between mother and fetus (Feldman, 2006) and continues to characterize relational events in the entire human lifespan. Although referable to a wide pattern of phenomena underpinned both by central and peripheral nervous system, the term physiological synchronization (PS) is mostly used in literature to denote the autonomic nervous system mutual modulations (Palumbo et al., 2017), of two or more people interacting [even without eye contact, e.g., only by hearing each other; Vanutelli et al. (2017)]. Even though PS seems to occur also during behavioral coordination, the great majority of studies are recently addressed to define the intriguing phenomenon of the co-occurrence of PS and emotional/affective attunement, happening even when interacting people are involved in minimal and not necessarily coordinated behaviors [e.g., eye gaze; Palumbo et al. (2017)].

The neurotransmittitorial system underpinning PS has never been investigated. Our idea is that oxytocin may be the eligible candidate to be investigated as a mediator of PS, on the basis of the following arguments: (a) Both PS and oxytocin release occur in the same affiliative processes; (b) Aggressivity and conflictual exchange are characterized by both PS and oxytocin release, an aspect that is only in ostensible contradiction with the previous one; (c) PS and oxytocin system functioning have compatible neural underpinnings; (d) PS and oxytocin have all shown an association with psychotherapy process and outcome.

### Oxytocin, Physiological Synchronization, and Affiliative Processes

Oxytocin and PS have both been previously and extensively associated to social and affiliative processes such those attachment system–related, and to affective empathy and social engagement. Namely, the link between oxytocin and attachment has already been established in the 1990's (e.g., Insel, 1997), with studies correlating oxytocin receptor polymorphism with attachment behaviors (Chen et al., 2011). Ham and Tronick (2009) associated the attachment system with synchronization patterns of child-caregiver dyads by demonstrating how attachment task of the still-face paradigm was characterized with high PS during reunion episode, in which mother and infant generate a new attunement. In clinical settings, the manipulation of the sense of attachment security in therapists produced an effect on the lag of the patients' and therapists' PS during clinical interactions (Palmieri et al., 2018). Similarly, speech markers identified as related to secure attachment were positively associated with patterns of high PS (Kleinbub et al., 2020a). Experimental studies have also demonstrated that long-term oxytocin administration enhances the experience of attachment between interacting adults (Bernaerts et al., 2017).

Both oxytocin and PS are significantly associated to empathy. A series of studies in psychotherapy research field, for instance, demonstrated a positive correlation between the amount of therapist-patient PS and the level of affective empathy (Messina et al., 2013; Kleinbub et al., 2019). As a whole, Palumbo et al. (2017) review highlighted that manifestations of PS in human interactions occur during shared experience marked by empathy and its validity as an objective index of empathic, affiliative phenomena, has been demonstrated (Kleinbub et al., 2019). Oxytocin release has also been found to be associated with affective empathy: studies indicate that dispositional empathy traits are sensitive to genetic variations of oxytocin receptor (Smith et al., 2014), and that oxytocin administration enhances the ability to correctly infer the emotional content of social stimuli (Hurlemann et al., 2010).

Social engagement was recently linked to PS as well as to oxytocin in mice (Kingsbury et al., 2019) as well as in humans, by demonstrating that a physiological biofeedback-based training can ameliorate people's ability to empathize with others (Gennaro et al., 2019; Kleinbub et al., 2020b). Oxytocin has also been suggested to mediate social engagement dynamics by facilitating trust and cooperation needed to adjust to new social groups (Anacker and Beery, 2013).

### Oxytocin, Physiological Synchronization, and Aggression

Oxytocin and PS exert similar effects during aggressive social interactions as those observed during social dynamics. For example, PS increases during marital conflicts (Timmons et al., 2015), and studies also demonstrate elevated oxytocin level in intimate partner violence (DeWall et al., 2014).

Such a paradoxical parallelism in seemingly opposite social states can be explained by Shamay-Tsoory and Abu-Akel (2016) Social Salience Hypothesis, a theoretical framework focusing on the effects of oxytocin on the salience of social cues. According to this hypothesis, oxytocin increases the sensitivity to social cues (Olff et al., 2013) depending on contextual and/or individual factors (Bartz et al., 2011). Following such a hypothesis, it can be suggested that both PS and oxytocin are socially and relationally dependent, thus exerting similar effects in either affiliative or aggressive states. Consistent with such a perspective, Feldman (2020) postulated that oxytocin and neural synchrony both function in the service of a superordinate meaning system which can affect not only in-group empathy, but also out-group derogation.

### Physiological and Anatomical Bases of the Physiological Synchronization and Oxytocin System

PS studies usually focus on autonomic nervous signals such as electrodermal and cardiac activity, and–less frequently–on respiratory activity or variations in body temperature (Palumbo et al., 2017). These physiological activations are regulated by sympathetic and parasympathetic branches mainly through Noradrenaline and Acetylcholine. These two branches are regulated by the paraventricular nucleus (Maejima et al., 2019) which contains Corticotropin–Releasing Factor neurons and oxytocinergic neurons, exerting opposite actions. The Corticotropin–Releasing Factor neurons increase the activity in the Hypothalamic-Pituitary-Adrenal axis and project to brainstem areas to increase the activity of the sympathetic nervous system. Oxytocinergic neurons decrease the activity of the Hypothalamic-Pituitary-Adrenal axis and of the sympathetic nervous system and increase the function of the parasympathetic nervous system in the brainstem.

Oxytocinergic nerves emanating from the paraventricular nucleus project to many areas, including areas known to modulate emotional functions such as the amygdala (Stoop et al., 2015), areas involved in pain control such as the periaqueductal gray and the spinal cord, and areas modulating incoming sensory information in the cortex, the last being a crucial aspect in the interpersonal exchange and hence in PS phenomena. Of note, oxytocin is not present in autonomic nerves—all oxytocinergic nerves originate in the hypothalamic paraventricular nucleus and supraoptic nucleus—but they are involved in the control of the autonomic nervous system through brainstem centers innervations. This association with areas in the brainstem is especially relevant to highlight the linkage between PS and oxytocin, as oxytocin activates sympathetic and parasympathetic branches by increasing overall autonomic control and regulation (Tracy et al., 2018).

Moreover, the central autonomic network (Benarroch, 1993), which includes higher central areas with remarkable mirror properties, has been recently described as being mediated by oxytocin (Festante et al., 2020). These areas include the anterior cingulate, ventromedial prefrontal and insular cortices, and should play an important role not only in behavioral synchronization but also in the synchronization of the function of the autonomic nervous system.

**Figure 1:**
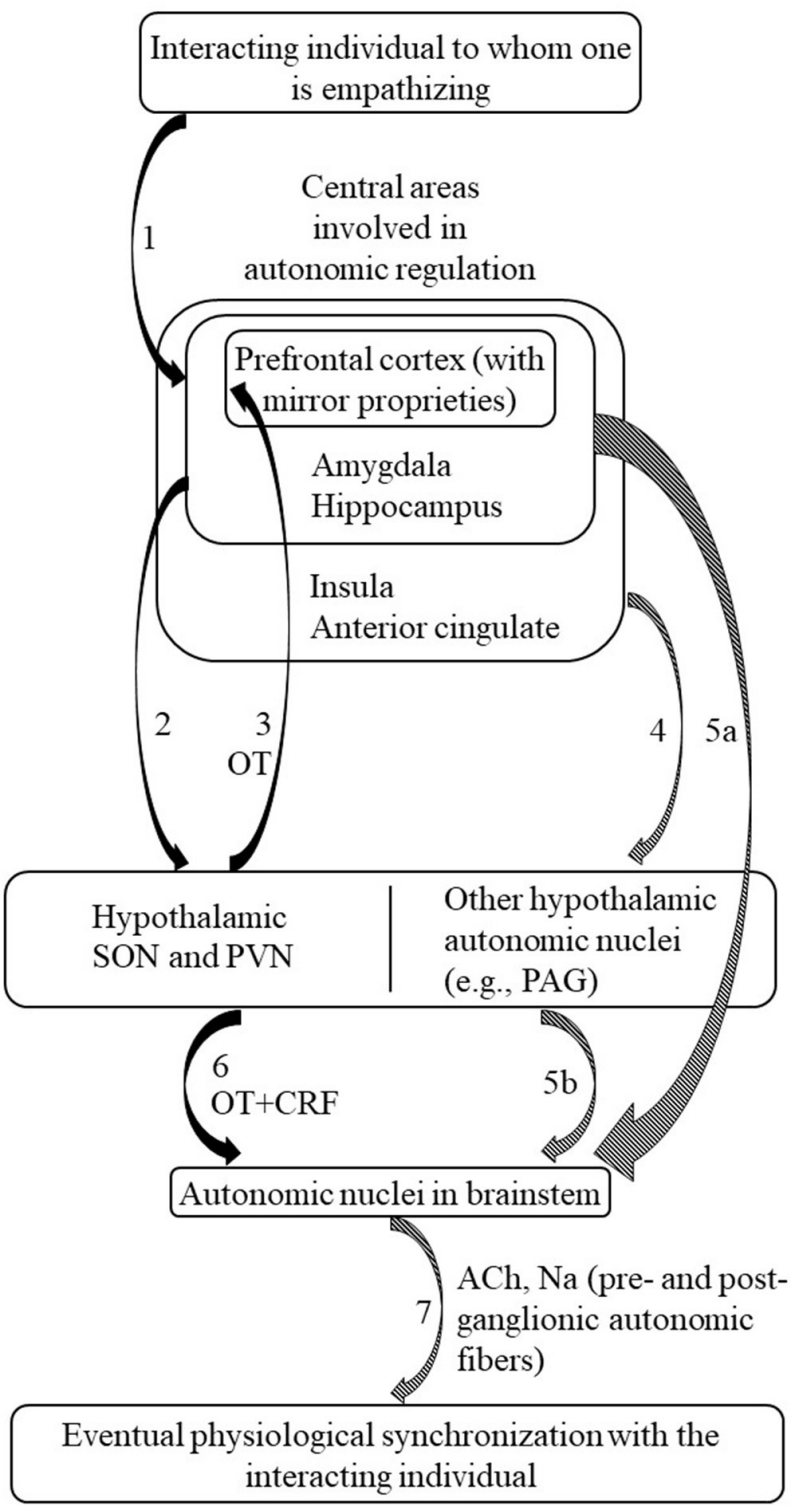
Hypothesized pathway of oxytocin mediation on the autonomic nervous system, in terms of physiological synchronization among interacting individuals. (1) The person to whom one empathizes (or to whom one is attached to/is socially engaged with/that is perceived relevant in ingroup-outgroup dynamics) impacts firstly on the brain areas involved in processing social environmental stimuli, i.e., the prefrontal cortex, amygdala, and hippocampus (Uvnäs-Moberg et al., 2005). (2) The prefrontal-amygdala-hippocampus complex influences oxytocin release in PVN and SON hypothalamic nuclei (Uvnäs-Moberg et al., 2005). (3) In turn, oxytocin released in PVN and SON influences the mirror system expressed in the prefrontal structures (Festante et al., 2020). Namely, this pseudo-circular feedback and feedforward system should facilitate mirroring, possibly by making the mirror neurons more sensitive. (4) CAN and its related paralimbic nuclei, including the prefrontal-amygdala-hippocampus complex, directly regulates hypothalamic autonomic nervous system nuclei (Benarroch, 2012). (5) The prefrontal structures and the amygdala-hippocampus complex directly influence the brainstem, thanks to mirror properties and the affective stimulus detection (5a); Autonomic hypothalamic nuclei project to autonomic brainstem nuclei (5b) (Benarroch, 1993). (6) Parallelly to direct autonomic efferences from hypothalamic to brainstem nuclei, oxytocin released from PVN and SON, together with CRF, modulates the action of brainstem autonomic nuclei (Geerling et al., 2010). (7) From the brainstem, sympathetic and parasympathetic nervous system branches are mediated by Acetylcholine e Noradrenaline, depending on form the autonomic branches and the preganglionic or post-ganglionic efferences. These cascade processes, as a whole, activate the peripheral nervous system physiological signaling, which is eventually able to synchronize with another one's physiology thanks to oxytocin mediation. ACh, Acetylcholine; CAN, Central Autonomic Network; CRF, Corticotropin–Releasing Factor; Na, Noradrenaline; OT, Oxytocin; PAG, periaqueductal gray; PVN, Paraventricular nucleus; SON, Supraoptic nucleus. Dashed arrows, actions that do not directly involve the oxytocinergic pathway.

Thus, the joint activation of both central and peripheral areas may produce synchronized dynamics during social interaction (Ramachandran et al., 2009; Palmieri et al., 2018; Kleinbub et al., 2019) in which the mirror neuron mechanisms should play an important role by picking up the intensity of autonomic nervous function from another individual via incoming sensory information. In the cortex, this information may in turn produce a synchronized response through oxytocin's indirect regulation of the peripheral, autonomic systems. In other words, there are links from these higher, anterior centers, to brainstem areas involved in the ongoing regulation of autonomic nervous tone, implicating mirror mechanisms triggering the coordination between own's and other individual's cues, thanks to mirror system sensitivity to subtle interactional aspects, such as facial expressions or vocal tone (Cacioppo et al., 2017). Figure 1 reports the hypothesized oxytocinergic pathway leading to PS.

### Physiological Synchronization and Oxytocin in Psychotherapy Settings

Synchronization between patient and psychotherapist is linked to beneficial psychotherapeutic outcomes (e.g., Ramseyer and Tschacher, 2014), as well to therapeutic alliance (Bar-Kalifa et al., 2019), and to empathy perceived by patient (Marci et al., 2007; Messina et al., 2013; Kleinbub et al., 2019). These kinds of findings led to a recent, dramatic increase of interest in PS in psychotherapy research (Kleinbub, 2017). On the other hand, oxytocin administration was also found to enhance therapeutic response in hypnotic-based settings (Bryant et al., 2012), and recent studies have also demonstrated that oxytocin synchronization predicted better therapeutic outcome (Zilcha-Mano et al., 2020). Oxytocin has also been suggested to exert effects on synchronization phenomena in psychotherapy settings. For instance, Ramseyer et al. (2020) investigated synchronization of spontaneous head and body movements between patient and therapist, and found that oxytocin administration prior to a psychotherapy session resulted in higher movements coordination, compared to placebo condition. Although synchronization in body movements does not always coincide with PS (Palumbo et al., 2017), these intriguing results encourage the efforts toward filling the gap in the research on the association between oxytocin and all human synchronization phenomena.

## Discussion

This contribution is aimed to encourage empirical investigation of the association between oxytocin and PS, mainly, but not only, in psychotherapy settings. Future studies should firstly consider the action mechanisms of endogenous vs. exogenous (usually intranasal) oxytocin administration, parallelly to PS detection in the interacting dyad, as these two forms of oxytocin present several differences. Endogenous oxytocin reaches specific brain areas provided with oxytocin receptors, while exogenous oxytocin does not induce all the natural effects of endogenous oxytocin, as circulating oxytocin does not pass the blood-brain barrier. On the other hand, exogenous oxytocin is more easily controlled–it has been used as an augmenting outcome factor in psychotherapy research since the early 2000's (Guastella et al., 2009)–whilst plasma and salivary oxytocin levels are more complex to be collected and interpreted, as different sources provide different levels of oxytocin, and do not necessarily reflect its concentration at nerve terminals or in the brain.

From PS perspective, the two most used indices, i.e., heart rate variability and electrodermal activity, should be distinguished as well, since they have substantial differences that need to be considered prior to experimental manipulation. The former is influenced by both the activity of the parasympathetic and the sympathetic branches and these components are inferred by *ad hoc* ratio (LF/HF ratio), whilst the latter is an index of pure sympathetic activity from which only indirectly one can infer the parasympathetic one (Cacioppo et al., 2016). Useful algorithms for PS in terms of dyadic electrodermal activity analysis are already available and can be utilized in such studies (e.g., Kleinbub, 2019).

Given these premises underlying the implications for methodological choices, a straightforward approach to investigate the compelling hypothesis of oxytocin mediation on PS would be to compare patients engaged in a brief psychotherapy in a double-blinded, placebo-controlled, randomized study, where oxytocin vs. placebo would be administered before each session. In both groups, pre-post therapy assessments should be carried out to evaluate therapeutic change, and patient's and therapist's physiological signals should be continuously and simultaneously measured. The main expectation would be that clinical dyads in which patients received oxytocin show higher average PS. Alternatively, a more naturalistic design would require only a single group, where endogenous oxytocin can be measured by assessing the levels of oxytocin in each member of the dyad. In such methodological settings, we would expect to find a positive correlation between oxytocin increase and amount of PS. Further studies should explore the physiological processes implying the role of mirror mechanisms in their potential explanatory route in the hypothesis of oxytocin as neurotransmittitorial mediator of PS.

Overall, the hypothesis of oxytocin as the likely mediator of PS, although we focused on psychotherapeutic relations as eligible experimental setting, is clearly extensible to all human interactions, and, like a lacking piece of a jigsaw puzzle, would help to converge many research findings and theoretical conception currently still fragmented. Scholars are hence encouraged to investigate the potential coupling of oxytocin and PS firstly, but not only, in psychotherapeutic settings, as social and wellbeing implications in terms of understanding human relations and shaping clinical interventions can be significant at many theoretical and pragmatic levels.

## Author Contributions

AP conceptualized the main idea. AP, EP, AG-G, and DTB drafted the manuscript. AP and DTB revised it critically. All authors approved the final version.

## Conflict of Interest

The authors declare that the research was conducted in the absence of any commercial or financial relationships that could be construed as a potential conflict of interest.
